# Case Report: Adrenocortical carcinoma in children—symptoms, diagnosis, and treatment

**DOI:** 10.3389/fendo.2023.1216501

**Published:** 2023-11-21

**Authors:** Estera Zagojska, Magdalena Malka, Adrianna Gorecka, Iwona Ben-Skowronek

**Affiliations:** Department of Paediatric Endocrinology and Diabetology, Medical University of Lublin, Lublin, Poland

**Keywords:** adrenocortical carcinoma, adrenocortical tumour, precocious puberty, Cushing syndrome - therapy, Cushing syndrome

## Abstract

Adrenocortical carcinomas are extremely rare in the paediatric population. Most of them are hormone-secretive lesions; therefore, they should be taken into consideration in a child with signs of precocious puberty and/or Cushing’s syndrome symptoms. Nonetheless, differentiation from benign adrenal tumours is necessary. We report a rare case of adrenocortical carcinoma in a girl and a literature review using the PubMed database. A four-year-old girl presented with rapidly progressing precocious puberty and signs of Cushing’s syndrome. Imaging of the abdomen revealed a large heterogeneous solid mass. Histopathologic evaluation confirmed adrenocortical carcinoma with high mitotic activity, atypical mitoses, pleomorphism, necrosis, and vascular invasion. After tumourectomy, a decrease of previously elevated hormonal blood parameters was observed. Genetic tests confirmed Li Fraumeni syndrome. Adrenocortical carcinoma should be suspected in children with premature pubarche and signs of Cushing’s syndrome. Diagnosis must be based on clinical presentation, hormonal tests, imaging, and histopathological evaluation. Complete surgical resection of the tumour is the gold standard. Oncological treatment in children is not yet well-studied and should be individually considered, especially in advanced, inoperable carcinomas with metastases. Genetic investigations are useful for determining the prognosis in patients and their siblings.

## Introduction

Adrenocortical tumours (ACT) including adrenocortical carcinomas (ACC) and adrenocortical adenomas (ACA) are rare in the paediatric population, with an incidence of 0.3–0.38:1,000,000, accounting for approximately 0.2% of all paediatric neoplasms (1–4).

ACT are more common in women, with a proportion of 1.4:1. This rate varies according to age, but remains higher in women (2, 4, 5), especially in malignant adreno-cortical carcinomas; however, there are certain subtypes of ACC, such as oncocytic, with no preference for any sex (6).

A much higher percentage of ACT is observed in Brazil, with an incidence of 6.2:1,000,000 in children <10 years (7), and for ACC, the incidence is 2.2:1,000,000 in the general population and 4:1,000,000 in children <10 years (8). This is due to the greater prevalence of the TP53 mutation, especially in industrial and urban areas (7).

The majority of ACT and ACC are sporadic; however, there is an increased risk of developing the tumour in patients with genetic syndromes such as Li Fraumeni syndrome, Beckwith–Wiedemann syndrome, multiple endocrine neoplasia type 1 (MEN-1), familial adenomatous polyposis, and other hereditary cancers (6, 9, 10).

Most ACT in the paediatric population are hormone-secreting lesions (1, 2). The hypersecretion of adrenocortical hormones leads to clinical manifestation, most commonly peripheral precocious puberty with virilisation and Cushing’s syndrome, and rarely, hyperaldosteronism (1, 2, 4, 5).

Non-functional ACT and ACC are much less common. The manifestation of adrenal tumours is non-specific, including abdominal or back pain, fatigue (1, 5), or acute abdomen (11, 12).

The differentiation between benign adrenocortical adenomas and ACC presents a challenge at every stage of the diagnostic process, including clinical symptom analysis, laboratory tests, imaging scans, and histopathologic evaluation (1, 2, 13, 14).

The only undisputed sign of malignancy is metastases. The most frequent sites of ACC metastases are the lung, liver, and retroperitoneal space (15). Metastatic disease is one of the most adverse, significative prognostic factors at the time of diagnosis—this raises the need for early diagnosis (1, 2, 13, 16).

The aim of this study was the presentation of a rare cancer patient and a review of the up-to-date knowledge of adrenocortical carcinoma and its diagnostics-therapeutics algorithm.

## Case presentation

A four-year-old female presented with rapidly progressive precocious puberty. Pubic hair was first observed approximately two months prior to presentation. Additionally, the child’s mother noticed intense perspiration and acne. The patient gained approximately 3 kg in one month and became apathetic.

The patient’s medical history was insignificant: second pregnancy, second delivery (natural delivery at the 39th week of gestation), Apgar score of 10, birth weight of 3,900 g, and birth length of 55 cm. Psychomotor development was normal. She had no chronic diseases, no allergies, or no operations in the past. Her diet was normal and physical activity was regular; however, she became less active in the weeks prior to presentation.

**Figure 1:**
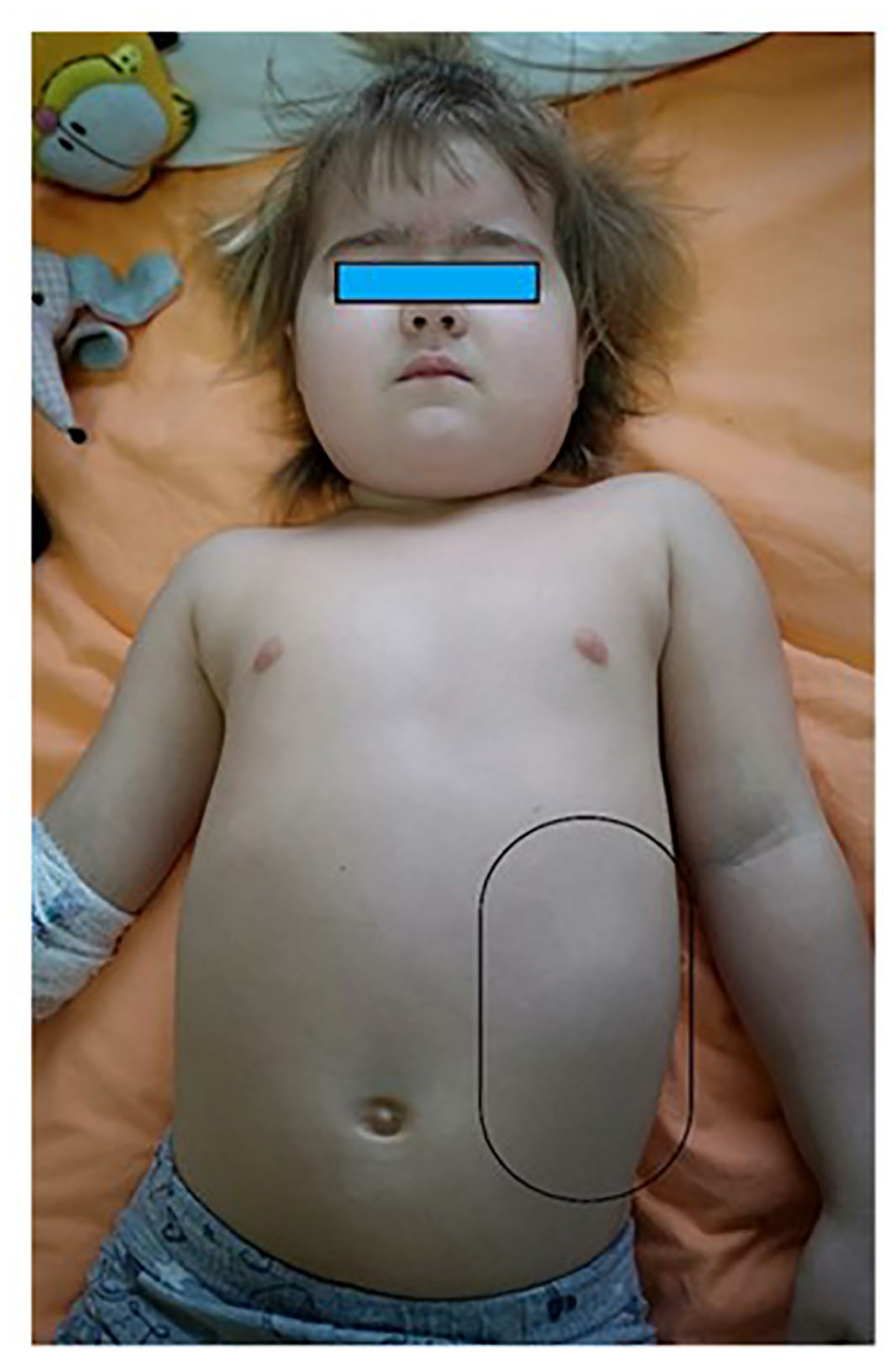
The patient’s appearance. A large mass is visible in the left abdomen.

On physical examination, the patient’s general condition was good, her vital signs were stable, her height was 110 cm (90th percentile), her body weight was 23.9 kg (>97th percentile), and she appeared overweight with an excess of visceral adipose tissue, a rounded face, and prominent, reddened cheeks. She also had acne (**Figure 1**).

The abdomen was asymmetrically distended and a firm, non-tender, and immovable mass was palpated on the left side. Physical examination also revealed pubarche, Th1 P3 A1 on the Tanner scale.

Abdominal ultrasonography (USG) revealed a large heterogeneous solid mass between the spleen and left kidney, measuring 121 x 100 x 141 mm, with several blood flow signals. The mass shifted and modelled surrounding organs. Additionally, a small round lesion of unidentified character was observed in the right lobe of the liver.

**Figure 2:**
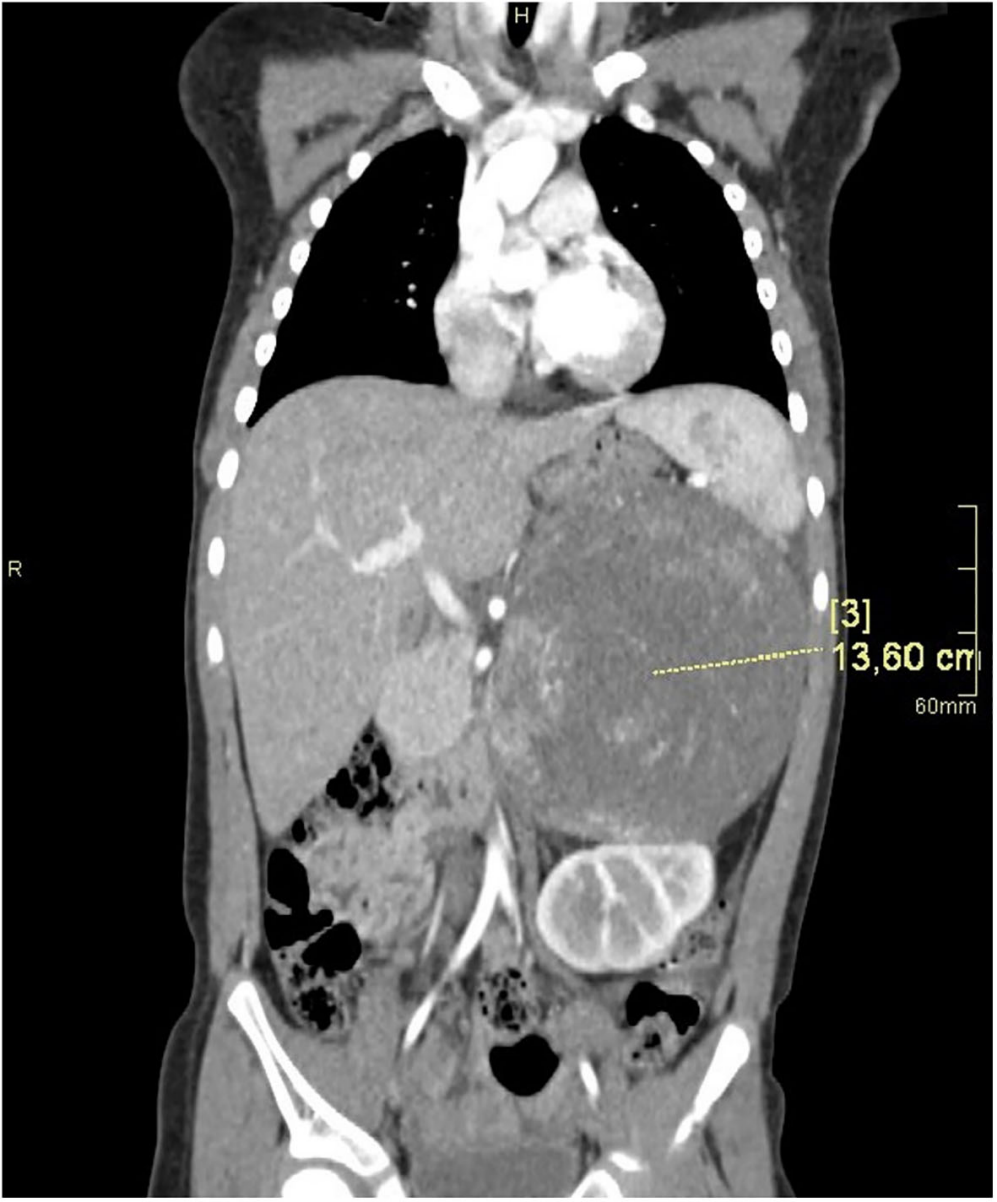
CT of the abdomen in the AP plane.

**Figure 3:**
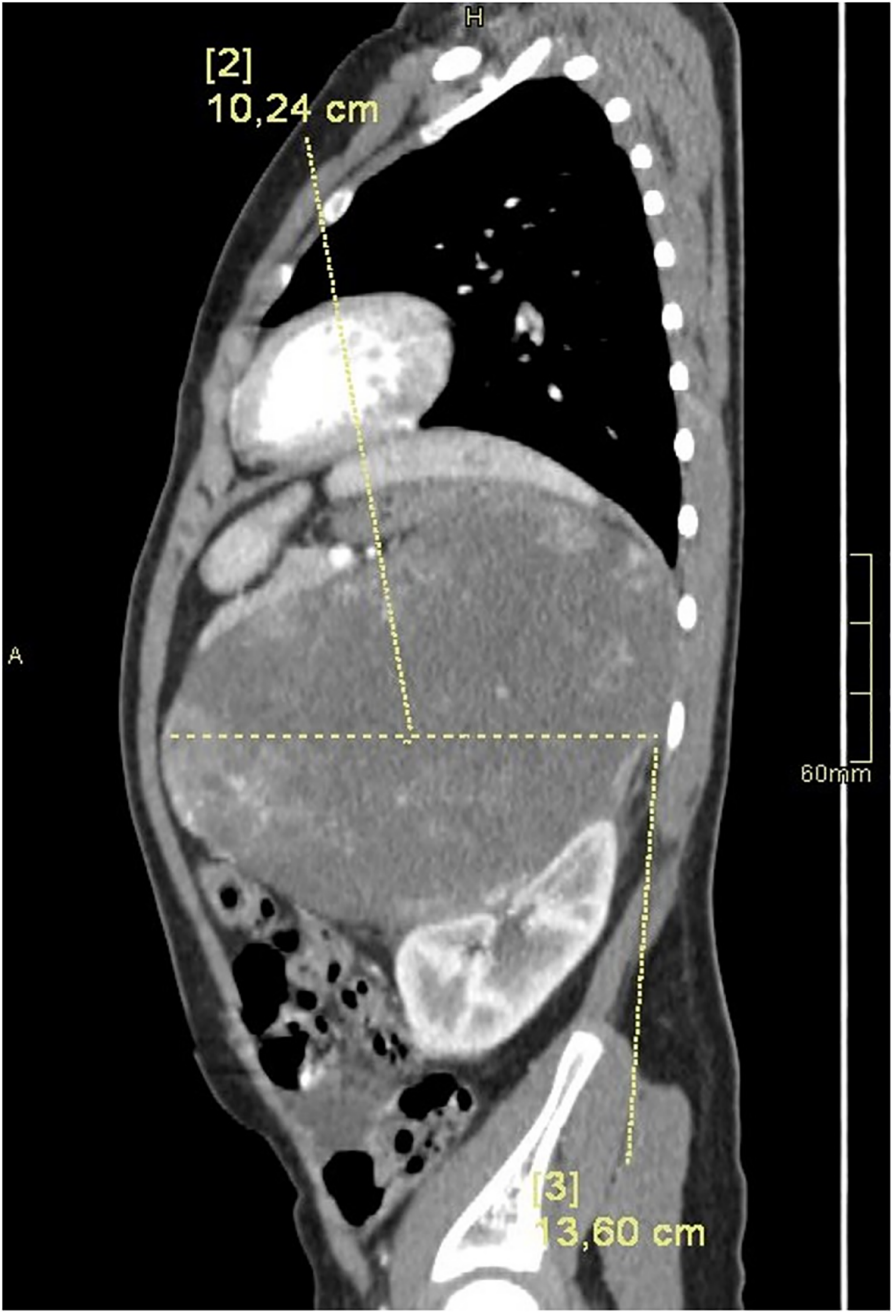
CT of the abdomen in the LR plane.

Computed tomography of the abdomen showed a large left suprarenal mass measuring left-right (LR) 10 cm; anterior-posterior (AP) 13.5 cm; and cranio-caudal (CC) 12 cm, with calcifications (**Figures 2**, **3**). The tumour compressed and shifted the tail of the pancreas, stomach, spleen, and left kidney. On post-contrast-enhanced CT there was a heterogenous enhancement in the lateral parts, with irregular low-density areas of tumour necrosis and lysis in the centre. A lesion measuring 11 x 13 mm, characteristic for angiomas, was found in the VI segment of the liver with contrast enhancement. Chest CT did not show any abnormalities.

The patient’s bone age was six years based on the Greulich-Pyle method.

Laboratory investigations are shown in **Table 1**.

**Table 1:** Laboratory investigations before and after tumour surgery.

Parameter	Result before treatment	Results after surgery	Normal ranges	Units
Cortisol 8:00 AM	22.52	12.86	6.2–19.4	ug/dl
Cortisol 11:00 PM	21.36	5.22	2.3–11.9	ug/dl
ACTH	3.82	32.8	7.2–63.6	pg/ml
24 hr urine Cortisol	184.80	104.1	4.3–176	ug/24 h
Steroid profile GC-MS	Typical for ACC secreting cortisol and androgens, showing intensive enzymatic abnormalities—deficiency of 3BHSD (massive excretion of DHA and its metabolites), deficiency of 11-B OH, 21-OH, and increased excretion of 6B-OHF	All steroids in normal range		
Testosterone	1,366.00	6.2	6–82	ng/dl
DHEAS	1,000.00	5.92	0.47–19.4	ug/dl
Androstenedione	>30	1.25	0.3–3.3	ng/ml
Oestradiol	<5	<5	6–27	pg/ml
Progesterone	2.30	0.99	0–0.99	ng/ml
17-OHP	25.11	0.5	0.2–0.9	ng/ml
LDH	3 184	423	0–615	U/l
NSE	77.03	12.8	0–16.3	ng/ml

ACTH, adrenocorticotropic hormone; GC-MS, gas chromatography-mass spectrometry, 3BHSD; 3B-hydroxysteroid dehydrogenase; 11B-OH, 11B hydroxysteroid dehydrogenase; 21-OH, steroid 21-hydroxylase; 6B-OHF, 6B-hydroxycortisol; DHA, dehydroandrostendion; DHEAS, dehydroepiandrosterone sulphate; 17-OHP, 17-hydroxyprogesterone; LDH, lactate dehydrogenase, NSE, neuron-specific enolase.

The girl was qualified for surgical intervention. Before surgery, she was prepared with hydrocortisone in high substitutive doses. The patient was referred to the Department of Surgery, The Children’s Memorial Health Institute, where she underwent radical tumourectomy, with resection of the left kidney and the lesion in the liver. After the tumourectomy, the high doses of hydrocortisone were continued. The perioperative period was complicated by lymphorrhoea, pancreatitis, pneumonia, left side pleural effusion, and gastrointestinal obstruction that required a second laparotomy. In the subsequent days, the patient improved. The substitution of hydrocortisone was decreased, and fludrocortisone was introduced orally.

Histopathologic evaluation of the tumour confirmed ACC with high mitotic activity, atypical mitoses, pleomorphism, necrosis, and vascular invasion. The structure of the left kidney was normal. No metastases were found in the local lymph nodes. The lesion in the sixth liver segment was confirmed to be a cavernous angioma.

In the post-operative follow-up, the patient remained under observation of the Department of Oncology in The Children’s Memorial Health Institute, Warsaw. In a control CT of the abdomen, chest, and pelvis, no lesions were observed. The control steroid profile showed normal daily excretion of androgens and 17-OH-progesterone metabolites.

At the request of her parents, the patient was transferred to the Department of Haematology, Oncology and Transplantology of the Children’s Hospital of Lublin to continue treatment. At this stage, no new symptoms presented. The development of the girl is normal: her Tanner scale is Th1P2A1. The second control steroid profile was normal.

Genetic investigations confirmed Li Fraumeni syndrome in the girl. There is a need to examine the patient’s siblings. The parents did not consent to oncological treatment of their daughter.

## Review

### Epidemiology and genetics

ACT, encompassing both benign and malignant lesions, are rare in the paediatric population, accounting for 0.2% of paediatric neoplasms. The literature reports the peak incidence of ACT in children at either <five years, or at a biphasic age distribution, with the first peak <five years and the second >10 years (1, 2, 4).

At the molecular level, two common aberrations are described—excessive expression of IGF-2 and constitutive activation of the signal route Wnt/β-catenin (4, 14, 17, 18). Identification of these aberrations correlates with a poor prognosis.

It was recently observed that germline EGFR variants (19) and vitamin D receptor hypermethylation and underexpression (20) may predispose infants to childhood ACC. High expression of Stathmin1 was also observed in paediatric adrenal tumours (21). Thus, genetic analysis in children with ACT is important, especially those with second primary cancer or a positive family history of malignancies (6, 9).

### Clinical manifestation

Clinical manifestation of ACT is different in children and adults. Adults present with non-specific symptoms more often than hormonal hypersecretion due to the tumour-mass effect (4), while clinical manifestation in children is usually a consequence of elevated levels of steroids and their precursors. Based on clinical observations, it is noted that ACT, especially ACC, secrete more than one hormone, resulting in mixed symptomatology. Non-secreting tumours are rare (1, 2, 4, 5).

The most frequent symptom of ACC in children is virilisation, which is characterised by precocious development of pubic or axillary hair, penis enlargement or erection, clitoris enlargement, accelerated growth velocity with high bone-age, acne, hirsutism, and voice changes. The second most common manifestation is Cushing’s syndrome, with symptoms such as central obesity, moon face, and hypertension (6, 9, 15, 19, 22, 23). There is a case described in the literature in which chronically elevated sex steroids and, therefore, activated GnRG pulse generator, lead to central precocious puberty (24). Hyperaldosteronism is uncommon (1, 5).

The appearance of precocious puberty should always prompt consideration of pathology within the adrenal cortex in the diagnostic process (16, 24–26). A sudden increase in body weight, especially in conjunction with precocious puberty, is also a red flag. It would seem that the combination of the above symptoms indicates suspicion of ACT, especially ACC (2, 24). There are several features that may suggest a malignant character of precocious adrenarche: age <four years, rapid progression, clitoral enlargement, significant acceleration of bone age paired with increased blood pressure, and features of Cushing’s syndrome (13, 25–27).

### Laboratory testing

Laboratory diagnostics of ACC are based on hormonal activity observed in over 90% cases. Clinical manifestation results from excessive secretion of steroids and their precursors by the tumour. The most common abnormalities are increased levels of DHEAS, androstenedione, testosterone, 17-OH progesterone, and 11-deoxycorticosterone (6, 24). Among the biochemical parameters, attention should be given to LDH, which might be useful in distinguishing between ACA and ACC. Although LDH is not a specific marker for ACC, an elevated level marks a risk of malignancy (2, 27). Other parameters such as cortisol level, DHEAS, testosterone, oestradiol, and aldosterone are not helpful in differentiating between ACC and ACA (2), although it was observed that testosterone, DHEAS, and 17A-hydroxyprogesterone are higher in childhood adrenal carcinoma than adenoma (27). Nevertheless, a laboratory workup is essential in every paediatric patient with ACT and should comprise circadian rhythm of cortisol secretion, 24 h urine cortisol excretion, testosterone, oestradiol, DHEAS, androstenedione, 17-OH-progesterone, aldosterone, PRA, and the steroid profile in urine. The recommended laboratory technique for steroids assessment is mass spectrometry (1, 5, 13). Bearing in mind the role of the TP 53 gene mutation and other genetic factors in ACT in children, it is advisable to also perform genetic tests (5).

### Imaging testing

Ultrasonography (USG) of the abdomen is the first stage of imaging in a patient with clinical signs of adrenocortical pathology. Computed tomography (CT) is the next step in the diagnostic process (1, 2) and it is the most commonly used imaging procedure to identify adrenal tumours, with the best cost-benefit ratio (28). It enables a more precise evaluation of the tumour than USG in terms of size, capsule, demarcation, areas of calcifications, necrosis, or bleeding. CT allows early diagnosis of ACT and preoperative staging to plan the operation, although it has limitations and cannot determine the benign or malignant character of the tumour.

According to Kuhlen et al. (29), however, abdominal magnetic resonance imaging (MRI) rather than CT should be preferred in addition to USG. It is suggested to perform USG, abdominal MRI, and chest CT (for the evaluation of lung metastases) in all patients, PET when there is a suspicion of high-risk ACC and bone scan or brain MRI when metastases are suspected.

Malignant tumours are usually bigger than benign ones; however, diagnosis cannot be based only on dimensions. Nonetheless, tumours ≤5 cm seem to be benign, while tumours ≥10 cm are more likely to be malignant, especially with a concomitant elevated LDH level (2).

On imaging, ACA have homogenous contrast enhancement and are well circumscribed, while ACC are usually characterised by a heterogenous structure with necrosis, haemorrhage, and calcifications. ACC can also invade local structures and vessels (30). The presence of metastases in imaging tests indicates ACC (1, 2, 13). Distant metastases most often invade the liver, lungs, kidneys, and bones; therefore, these areas need to be radiologically examined (31) by conducting pelvis and chest CT (1, 2, 29). FDG-PET is also used to identify metastases (29–31).

Tumour biopsy is not recommended because resection is a basis of treatment, and tumour rupture and spillage during biopsy worsen the prognosis (31).

### Histopathological examination

There is no single histopathologic trait that can reveal the malignant character of adrenal tumour, apart from invasion of nearby tissues. Nonetheless, architectural disarray, reticulin framework disruption pleomorphic, and large, clear, and granular or eosinophilic neoplasm with intranuclear inclusion and nuclear atypia may help differentiate between benign and malignant adrenal tumours and are indications of ACC rather than ACA (32).

Children with ACT have better prognosis than adults (33); however, histopathologic criteria predicting tumour behaviour in adults are unreliable in children (34). The most prevalent systems in differentiating between benign and malignant ACT are the Weiss scale, the modified Weiss scale, and the Wieneke index (1). Nevertheless, histopathological criteria that would permit unequivocal distinguishment between ACC and ACA remain difficult to establish (2). The rarity of ACT results in limited diagnostic experience. The Weiss scale and its modified version evaluate microscopic features and are also used in adult patients. The Wieneke index, preferred in the paediatric population, includes both macro- and microscopic features. Such divergence was less often noted when the Wieneke index was applied, suggesting the higher validity of this tool (1, 35, 36). Wieneke’s criteria for malignancy (36) are as follows:

Tumour weight >400g
Tumour size >10.5cm
Extension into periadrenal soft tissues and/or adjacent organs
Invasion into vena cava
Venous invasion
Capsular invasion
Presence of tumour necrosis
>15 mitoses per 20 high power field (400X)
Presence of atypical mitotic figures

According to Wieneke’s criteria, it is possible to determine the prognosis outcome of the patient. Two criteria indicate a benign long-term clinical outcome, three criteria indicate an intermediate/atypical/uncertain malignant potential while four or more criteria indicate a poor clinical outcome. (4, 36).

It was observed that the cellular proliferation index Ki-67 was significantly different between ACC and ACA, with a mean level of 30.2% (range 7–80%) and 9.9% (range 2–20%), respectively (27). In another study (37), it was observed that the Ki-67 labelling index of paediatric adenomas and carcinomas was much higher than in adult adrenal tumours and that a KI-67 index ≥15% could be used to presume poor outcome in the paediatric population. Currently, research is in progress on the usefulness of molecular biology techniques such as DNA methylation analysis for differentiation between benign and malignant ACT (14).

Fang et al. suppose that ACC development may depend on intracellular communications mediated by miRNA and mRNA (38).

### Prognosis

According to the European Network for the Study of Adrenal Tumours (ENSAT), there are four stages of ACC. Stage I and stage II are strictly localised tumours with a size of ≤5 or >5 cm, respectively. Stage III is characterised by infiltration of surrounding tissue, positive regional lymph nodes, or a tumour thrombus in the vena cava and/or renal vein. Stage IV is defined by the presence of distant metastasis (1, 4). Stage I and II, age <4 years, smaller tumour size, and virilisation as the only symptom of childhood ACC are correlated with better prognosis (1, 23, 39, 40). It seems that the risk of recurrence is directly proportional to the size of ACT (1, 4, 5). The difference in the survival rate between a Weiss score >6 and ≤6 is also significant—the higher score, the worse the prognosis (15, 37). A similar observation was made for the Wieneke score (37). According to the National Cancer Institute (NCI) PDQ cancer information summary on childhood ACC, unfavourable prognostic factors include large tumour size, older age, incomplete resection, microscopic tumour necrosis, and metastatic disease (41).

### Treatment

Complete surgical resection is the mainstay in ACT treatment as it enables full control of the disease and satisfactory clinical effects, especially in cases that are not advanced (1, 13, 42–44). In some cases, preoperative management with ketoconazole is reported, especially in hypercortisolism (42–44).

In malignant adrenal tumours, open surgery rather than laparoscopic should be prioritized (31). Increased hormones levels can decrease even after 24 h post-surgery (6). Corticosteroids should be supplemented both in the perioperative (31) and the post-operative period (24, 31) to avoid adrenal insufficiency due to a rapid decrease in hormone production. A very important procedure is substitutive treatment with hydrocortisone before and after surgery in patients with very low levels of ACTH as a result of overproduction of tumour steroids and blockade of ACTH secretion from pituitary glands. After surgery, the substitutive doses of hydrocortisone are reduced, and the addition of fludrocortisone is necessary for good electrolyte balance (42–44).

The effect of adjuvant chemotherapy in treating ACC is not satisfactory; adrenal cancer cells are resistant to common drugs. Mitotane, an inhibitor of the adrenal cortex, is commonly used in adult patients with ACT (3, 18, 42, 44). Due to its cytotoxic effect on adrenocortical cells, it inhibits adrenal steroidogenesis and decreases the risk of recurrence in adults (35, 42, 44). Experience with using mitotane in children is limited and its effect on the paediatric population is not yet well established; therefore, an individualised approach to each patient should be taken into account. Mitotane is mostly administered in inoperable tumours, tumours with positive margins, or advanced tumours with metastases (39), although there are studies describing mitotane usage in earlier stages of ACC (1, 15). There are cases of patients treated with mitotane who ended up with stable disease or complete remission (11, 15), as well as those who had poor prognosis or died as a result of the disease, despite chemotherapy (15, 22, 45–47). In cases of stable disease course, monotherapy with mitotane only may be used, while in aggressive ACC with metastases, the treatment combines both mitotane and cytostatics (42), mostly etoposide, doxorubicin, and cisplatin (EDP).

Toxicities related to combined therapy with mitotane and chemotherapy may lead to discontinuation of treatment (1, 4, 5, 48, 49).

During mitotane treatment, substitutive doses of hydrocortisone should be 2–3 times higher than those used for congenital, adrenal hyperplasia (CAH), and in some patients, fludrocortisone treatment for control of electrolytes is necessary. Serum mitotane evaluation should also be reported as well as the dose, usually every 15 days (42, 44, 47).

ACC seems to be sensitive to radiotherapy (RT). Adult patients who received RT as an adjuvant treatment had higher overall survival (p=0.004) than patients treated with surgery alone (50). Wiegering at al (51). analysed cases of children with ACC, in whom RT was administered. A systematic review shows that the majority of patients receiving RT were stage 2, although the treatment was also performed in stage 1, 3, and 4 children.

Currently, the role of immunotherapy in ACC treatment in adult patients is being investigated. The potential use of immune checkpoint inhibitors, such as pembrolizumab, which has a favourable safety profile and good tolerance in initial evaluation in adult patients (43, 52, 53), is noteworthy. The study of Iodine-131 Iodometomidate (131I MTO) targeted radionuclide therapy (54) and Yttrium-90/177Lu-DOTATOC in somatostatin expressing tumours (55) is promising. According to Akinkuotu et al. children with ACC had better survival than adults. Factors independently associated with worse survival included older age, metastatic disease, and receipt of lymph node surgery (56).

## Conclusions

ACC should be suspected in children with premature pubarche and signs of Cushing’s syndrome. For diagnosis, hormonal tests and imaging (especially USG and CT) are necessary. The surgical treatment of ACC is the gold standard, and oncological treatment should be individually considered as the second adrenal gland may be destroyed. Genetic investigations are useful for determining the prognosis in patients and siblings.

## Data availability statement

The raw data supporting the conclusions of this article will be made available by the authors, without undue reservation.

## Ethics statement

The studies involving humans were approved by Ethical Committee of Medical University in Lublin. The studies were conducted in accordance with the local legislation and institutional requirements. The human samples used in this study were acquired from a by-product of routine care or industry. Written informed consent for participation was not required from the participants or the participants’ legal guardians/next of kin in accordance with the national legislation and institutional requirements. Written informed consent was obtained from the minor(s)’ legal guardian/next of kin for the publication of any potentially identifiable images or data included in this article. Written informed consent was obtained from the parents of the child for the publication of this case report.

## Author contributions

EZ, MM, AG, and IB-S made substantial contributions to the conception, design, and acquisition of data, drafting of the article, giving final approval of the version to be published, and agreeing to be accountable for all aspects of the work in ensuring that questions related to the accuracy or integrity of any part of the work are appropriately investigated and resolved. IB-S critically revised the paper for important intellectual content. All authors contributed to the article and approved the submitted version.
